# Te-based chalcogenide materials for selector applications

**DOI:** 10.1038/s41598-017-08251-z

**Published:** 2017-08-14

**Authors:** A. Velea, K. Opsomer, W. Devulder, J. Dumortier, J. Fan, C. Detavernier, M. Jurczak, B. Govoreanu

**Affiliations:** 1 Imec, Kapeldreef 75, 3001 Heverlee, Belgium; 2 Ghent University, dept. Solid State Sciences, Krijgslaan 281 (S1), 9000 Ghent, Belgium; 3 National Institute of Materials Physics, Atomistilor 405A, P.O. Box M.G. 7, Magurele, 077125 Ilfov Romania

## Abstract

The implementation of dense, one-selector one-resistor (1S1R), resistive switching memory arrays, can be achieved with an appropriate selector for correct information storage and retrieval. Ovonic threshold switches (OTS) based on chalcogenide materials are a strong candidate, but their low thermal stability is one of the key factors that prevents rapid adoption by emerging resistive switching memory technologies. A previously developed map for phase change materials is expanded and improved for OTS materials. Selected materials from different areas of the map, belonging to binary Ge-Te and Si-Te systems, are explored. Several routes, including Si doping and reduction of Te amount, are used to increase the crystallization temperature. Selector devices, with areas as small as 55 × 55 nm^2^, were electrically assessed. Sub-threshold conduction models, based on Poole-Frenkel conduction mechanism, are applied to fresh samples in order to extract as-processed material parameters, such as trap height and density of defects, tailoring of which could be an important element for designing a suitable OTS material. Finally, a glass transition temperature estimation model is applied to Te-based materials in order to predict materials that might have the required thermal stability. A lower average number of *p*-electrons is correlated with a good thermal stability.

## Introduction

Emerging resistive switching (RS) non-volatile memory (NVM) technologies, such as resistive random access memory (RRAM) or phase change memory (PCM), are promising candidates for the next generation of advanced data storage applications^1, 2^. They have a number of advantages that could overcome the limitations of current memory technologies, such as scalability, non-volatility, high switching speed, energy efficiency and ease of fabrication^3–5^. Advanced data storage applications require NVM devices to be packed densely in vast cross-bar^6^ memory arrays to enable the storage of many terabytes of data, filling in the existing gap in the memory hierarchy. Accurate read and write operations in cross-bar arrays need high non-linearity in the current-voltage characteristics, to access a subset of the array. The currents passing through the selected cells have to be far greater than the residual leakage currents through the non-selected cells. A major bottleneck in the achievement of high density RRAM/PCM arrays is the lack of a high performing selector^7^ device in series with each memory element that allows for accurate information storage and retrieval, by suppressing parasitic currents, through highly nonlinear current-voltage (IV) characteristics, while enabling a sufficiently high drive current for the operation of the memory element.

There are several requirements^8^ that a selector device needs to meet. To first order, and first of all, it should provide the necessary current density in the ON-state to program and erase the memory element of the 1S1R cell. A switching current of only 10 μA, for a memory cell scalable to F = 10 nm (F being the feature size), requires a matching selector to enable a 10 MA/cm^2^ drive current. The leakage current should be low for the unselected cells, translating to a high IV nonlinearity. Half-bias voltage nonlinearity (NL_1/2_), defined as the ratio between the current at the operating voltage (i.e. the drive current) and the current at half of this voltage, should satisfy NL_1/2_ > 10^4^ or even higher, closely depending on the size of the array to be achieved^7^. Bidirectional selector IV nonlinearity is necessary whenever the operation mode of the memory element requires bipolar voltage application (which is for RRAM). Besides, voltage compatibility with the memory element is required and the selector should not limit the performance of the memory element. For this, it needs at least the same switching speed and endurance.

An important engineering requirement for OTS materials is to be back-end-of-line (BEOL)-compatible. The selector devices should be able to withstand 400 °C for up to 2 h, in order to account for the processing needs of the memory elements and wiring levels fabricated above them. In consequence, materials with high crystallization temperatures are necessary in order to increase the thermal stability.

Selector implementations are divided in several categories: Si-based devices such as transistors or diodes^9–11^, oxide-based diodes^12–15^, volatile switching selectors such as metal to insulator transition (MIT)^16, 17^ or ovonic threshold switches (OTS)^18, 19^ and mixed electronic and ionic conduction (MIEC)^20, 21^ selectors in Cu-based materials. OTS have low transition temperatures and usually cannot withstand the thermal budget for BEOL integration, although the early work of Kau, D. *et al*.^22^ showed a successful 1S1R cell integration. OTS^23^ is a volatile non-linear electrical characteristic of a two-terminal switching device in amorphous chalcogenide materials. The device can rapidly swap from a high resistive state (HRS) to a low resistive state (LRS) by applying a voltage that exceeds the threshold voltage. This state is maintained as long as a minimum holding voltage is applied to the device, otherwise it switches in HRS. The difference in resistance between HRS and LRS can be as high as six orders of magnitude. This unique property makes chalcogenide materials convenient for selector applications^24–29^.

In recent years, various models attempting to explain the switching phenomenon have been developed, such as Poole-Frenkel ionization, field-induced delocalization of tail states, space-charge limited currents, optimum channel hopping in thin films, optimum channel field emission, percolation band conduction, transport through crystalline inclusions^30^ or thermally-assisted threshold switching^31^.

A representative example of non-purely electronic model is the unified model of nucleation switching^32^. It provides a mechanism which explains the switching in both PCM and OTS memories, when the electric bias determines the formation of conductive filaments. The switching phenomenon is understood as the electric field induced nucleation of needle-shaped particles. The energy gained from the dipole moments of the particles exceeds the surface energy lost because of their non-spherical shape. This self-consistent model allows one to compute the threshold and holding voltages as a function of material parameters and device thickness.

On the other hand, many purely electronic models used to explain the non-linearity of subthreshold conduction in OTS are based on the Poole-Frenkel (PF) mechanism^33, 34^. The electric field increases the emission probability of charge carriers trapped in a Coulomb potential of a defect state in the bandgap. The energetic barrier for emission is lowered by the electric field and the emission occurs. Due to this lowering, the number of charge carriers emitted into the band increases and thus the conductivity rises. As shown in ref. 30, there are three conduction regimes in the subthreshold region, dependent on the applied field. The first one is the ohmic regime present at low fields (*F* < 10^3^–10^4^ V/cm), followed by a non-ohmic regime (*F* > 10^4^ V/cm), where *lnI* ~ *V* or *lnI* ~ $\sqrt{V}$ and an exponential regime at high fields with *lnI* ~ *V* ^2^. In the present study we have employed a model based on PF mechanism for the analysis of as-processed samples, as it allows an easy computation of the physical quantities which we are interested in.

Numerous physical processes and materials properties need to be taken into account when designing OTS based selectors^35^. A systematic approach similar to the one for phase change materials (PCM), where a map^36^ has been developed that offers some hints of where to look for suitable compositions, is still missing. The map is based on two bond orbital characteristics, namely hybridization and ionicity. The ionicity measures the difference in energy of the *p* orbitals between two atomic sites and is therefore a measure of the *p* state electronegativity. The hybridization measures the average *s-p* splitting between the two sites. PCM are situated at low ionicity and moderate hybridization, the bonds have a covalent character and they are not very strong, so they can be rapidly rearranged in order to obtain fast crystallization.

There were several goals that we had in mind when designing this study. The first one was to extend the PCM map to OTS compounds and use it to search in a systematic way for new selector materials. Secondly, to select several materials from this map and test its validity. Thirdly, to understand the interdependence between material properties and device features, governing the electrical behaviour of OTS materials in selectors, in order to be able to improve the OTS effect. Lastly, to use the extended map to predict new materials exhibiting OTS and to reliably estimate their thermal stability.

## Results and Discussion

**Figure 1:**
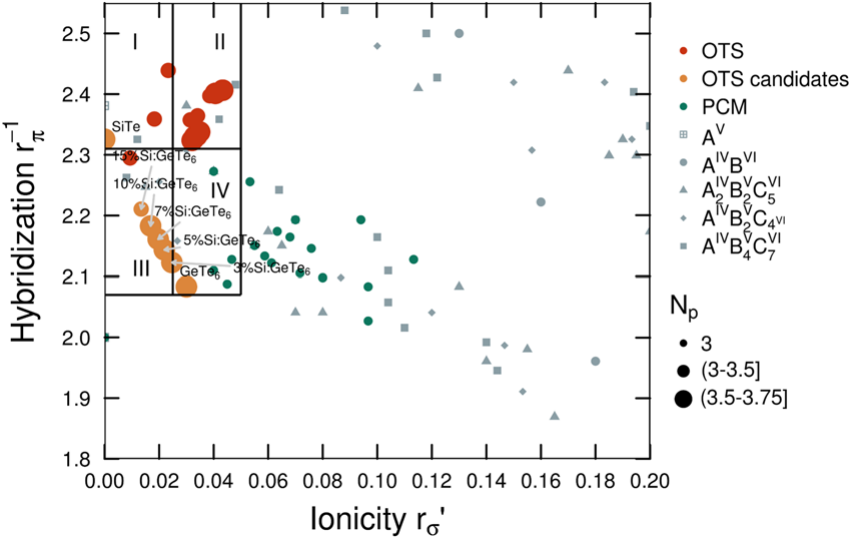
A map for OTS materials. In addition to PCM and OTS, other compositions such as group V elements (crossed squares), binary A^IV^B^VI^ compounds (circles) and ternary alloys with different compositions including A^IV^_2_B^V^_2_C^VI^_5_ (triangles), A^IV^B^V^_2_C^VI^_4_ (diamonds) and A^IV^B^V^_4_C^VI^_7_ (squares) from ref. 36 are shown. Areas highlighted in the plot, denoted as I, II, III and IV, are just for visualization purposes, splitting the area in 4 equal zones with high probability of finding OTS materials. Based on this splitting, we observed that most of the OTS materials reported by Ovshinsky^39^ lie in zone II. The model is not sensible to anion to cation ratios in binary chalcogenides (i.e. it cannot distinguish between GeTe and GeTe_6_, they lie in the same position on the map), but it discerns between anion to anion or cation to cation ratios (i.e. it can discriminate between Ge_2_Sb_2_Te_5_ and GeSb_2_Te_4_). Yellow dots are materials investigated in this study and they will be discussed in detail in the article.

An understanding of the relationship between stoichiometry, structure and physical properties of PCMs was proposed by Lencer *et al*.^36^. By plotting the hybridization as a function of ionicity for binary A^IV^B^VI^ and ternary A^IV^_2_B^V^_2_C^VI^_5_, A^IV^B^V^_2_C^VI^_4_ and A^IV^B^V^_4_C^VI^_7_ alloys, they observed that phase change materials (green dots in Fig. 1) are clustering in a certain area on the graph and they called it a treasure map for PCM materials. In order to check if the same methodology is applicable to OTS materials, we compute the two bond orbital coordinates using the orbital radii reported by Chelikowsky and Phillips^37^ for all Te-based OTS compositions that we could find reported in literature^27, 29, 38–40^, and we built a similar plot (red dots Fig. 1). The map from ref. 36 was designed for compositions with an average number of *p*-electrons (*N*_*p*_) equal to 3, which we expand and improve by the addition of data with various *N*_*p*_s and use it as an additional dimension (Fig. 1). The entire dataset of materials together with their computed values for hybridization, ionicity and *N*_*p*_ is listed in the Supplementary table ST1. Surprisingly, OTS materials are also clustered in a small area defined by lower ionicity and higher hybridization compared to phase change materials, indicating that they are likely to form more directed covalent bonds that will lead to a slower crystallization. This makes sense since in OTS the crystallization should be delayed as long as possible (which is also why many quaternary chalcogenides were investigated in the early days of OTS^24^). The rigidity of the structures is also explained by the slight increase in the average number of *p*-electrons, since bonding is primarily promoted by the *p*-electrons. We can observe that there is an area of low hybridization (zone IV) where we can find materials from binary Ge-Te system, but also alloys from Ge-Sb-Te, Ge-In-Te and Si-Sb-Te systems. The third zone is dominated by the Si-Ge-Te system, whereas in the first zone we can find mostly Si-As-Te and Sb-As-Te materials. Ge-As-Te based OTS materials are mostly placed in zone II, but for simplicity As was not used in our study.

**Figure 2:**
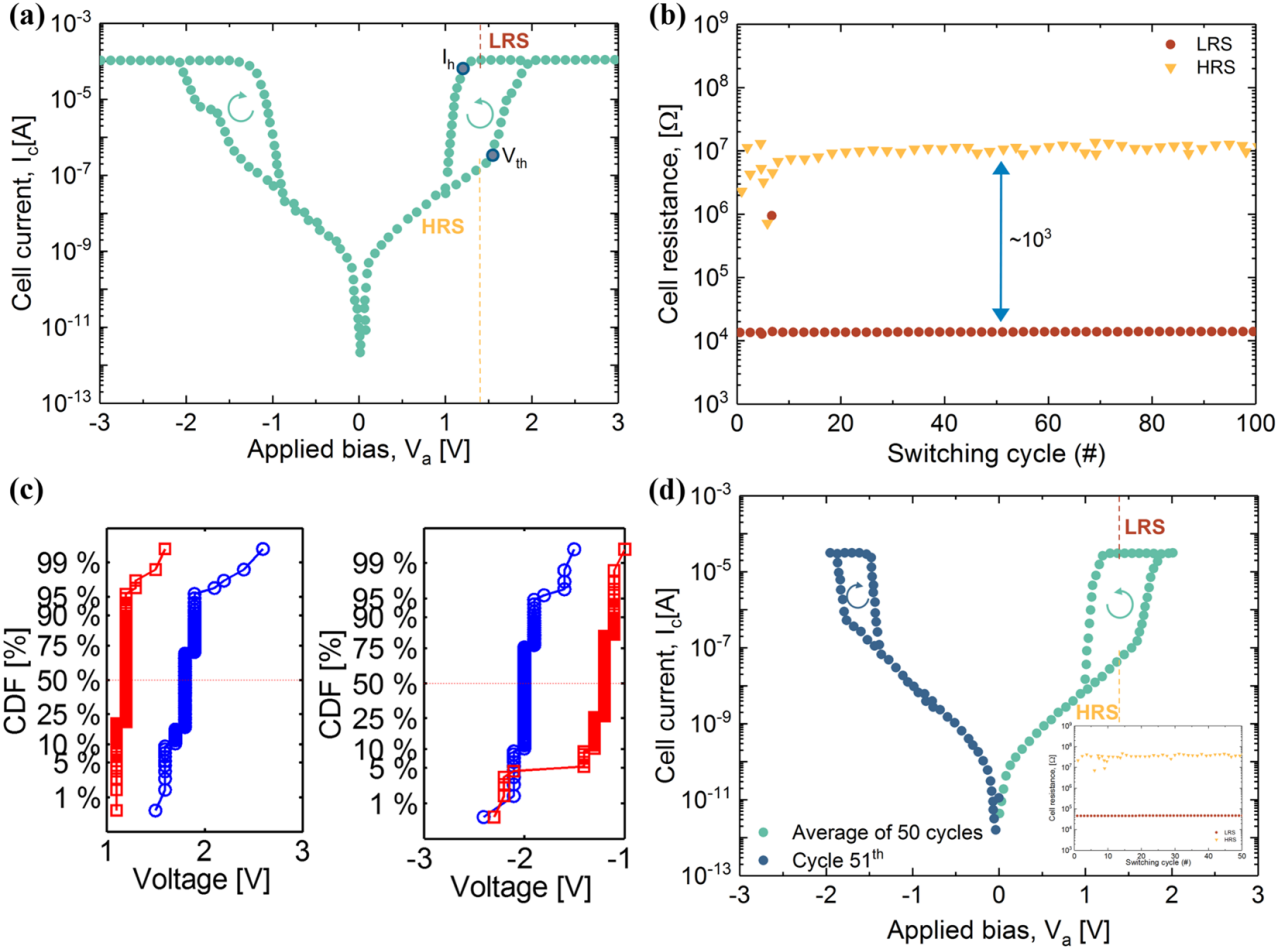
GeTe_6_ switching characteristics for a 135 nm device. (**a**): Average of 100 DC alternate polarity cycles. (**b**): Cell resistance in both HRS and LRS after each cycle. The reading voltage was 1.4 V (as suggested by the dashed red and blue lines). (**c**): Distribution of *V*_*th*_ (blue) and *V*_*h*_ (red) for both positive and negative polarities. (**d**): Average of 50 single polarity (positive) sweeps followed by a negative sweep. Inset: ON/OFF resistance ratio.

**Figure 3:**
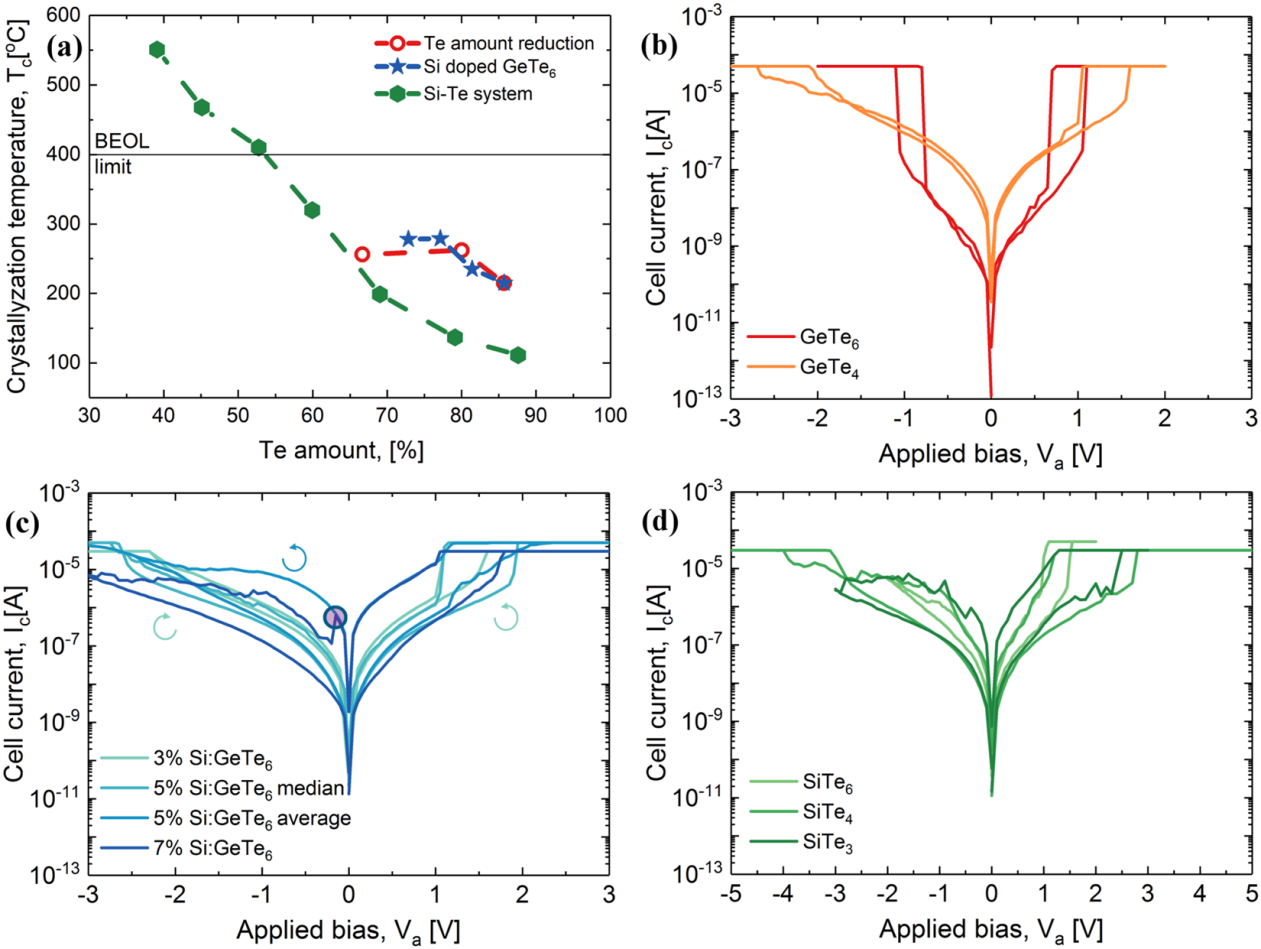
Thermal stability and electrical behaviour for (i) Tellurium amount reduction; (ii) Si doping; and (iii) Replacement of Ge with Si. (**a**): Crystallization temperature variation in all three cases. Typical electrical characteristics of 335 nm devices for (**b**): Ge-Te system; (**c**): Si doped GeTe_6_; The switching direction (‘set’) is given only for 3%Si:GeTe_6_, the rest of the compositions follow this direction for the positive sweeps, whereas for the negative sweeps at 5%Si: GeTe_6_ the direction changes from ‘set’ to ‘reset’; and (**d**): Si-Te system. Each curve from (**b–d**) is the median of 25 sweeps. 30 µA and 50 µA compliance currents were used.

We will subsequently sample several compositions from different zones on the map for OTS materials and test their suitability for selector applications, paying special attention to the question of whether simple binary chalcogenides can offer both electrical threshold switching and thermal stability. GeTe_6_^41^ is an eutectic point in the Ge-Te phase diagram reported as a good glass former^42^, with hundreds of microseconds long crystallization time. Figure 2(a) shows a characteristic OTS behaviour, averaged over 100 alternating positive and negative polarity DC sweeps. The maximum current density exceeds 3.3 MA/cm^2^ for devices of 55 nm size and the half bias nonlinearity is about 3 × 10^3^ (the current density is estimated with the external compliance used to limit the threshold switching in a voltage-controlled DC sweep). Almost three orders of magnitude contrast between LRS and HRS (Fig. 2(b)) for a reading voltage of 1.4 V, is observed. The distributions of holding and threshold voltages on both polarities for all the cycles are presented in Fig. 2(c). We have a symmetric behaviour with close median values for both positive and negative polarities and low variability with the threshold voltage of 2 V and holding voltage of 1.2 V. In order to prove that the switching is volatile (and we do not need alternate polarity sweeps to set and reset the devices - as in the case of RRAM devices), we performed 50 consecutive sweeps of positive polarity (Fig. 1(d)). We can observe that the switching performance is similar with the previous case. Different values of the compliance current (100 µA for Fig. 1(a) and 30 µA for Fig. 3(a)) show that compliance currents smaller than 100 µA have no trivial effect on the switching characteristics, at least for a short number of cycles (100 cycles). Recent studies on GeTe_6_^29, 43^ also showed OTS behaviour with a threshold voltage of 1.6 to 2.4 V, depending on the measurement type (AC or C-AFM) or thickness of the films (20 or 190 nm) and a holding voltage of 0.7 V. The results are comparable to our findings.

To study the thermal stability of GeTe_6_ (Fig. 3(a) and Supplementary Fig. S7(a)) we start with an amorphous film. Te crystallizes first above 200 °C, confirmed by the appearance of hexagonal Te (101) and (110) diffraction peaks. It is followed by rhombohedral GeTe crystallization at 300 °C ((024) and (220) peaks are noticed in the diffractogram). We might also expect phase separation due to atomic species diffusion during crystallization^42^. A large quantity of Te and GeTe melts just below 400 °C as suggested by the disappearance of Te crystalline peaks. Regarding the thermal stability, for GeTe_6_^44^ was observed a double exothermic transformation in DSC measurements attributed to Te segregation and crystallization at 210 °C followed by GeTe crystallization at 230 °C, similar to our results. For GeTe_4_, both Te and GeTe crystallites were seen in samples annealed at 210 °C, whereas GeTe_2_^45^ was found to crystallize at 240 °C. This means that, even if GeTe_6_ displays competitive electrical performance figures as a selector device, it unfortunately does not have the necessary thermal stability for BEOL CMOS integration.

Next, our efforts were concentrated on improving the thermal stability. We used three routes: (i) Te amount reduction from GeTe_6_ towards GeTe, since we observed that one of the reasons for low thermal stability is the crystallization and melting of Te; (ii) Si doping (up to 15%), because Si-Te bonds are stronger than Ge-Te bonds (the enthalpy of atomization for Si-Te bond is 215 kJ/mol, whereas for Ge-Te is 192 kJ/mol^46^), thus we expect an increase in the crystallization temperature; and (iii) completely replace Ge by Si and test the Si-Te binary system.

The effect of Te amount reduction is as expected (Fig. 3(a) and Supplementary Fig. S7). We get an increase of Te crystallization temperature from 215 °C to 260 °C, when we reduce Te from 85% to 80% and then it saturates, since now the bottleneck is the crystallization of GeTe which cannot be avoided. The melting temperature is unaffected by Te reduction and it stays at 375 °C. Regarding the electrical behaviour (Fig. 3(b)), by reducing Te from GeTe_6_ to GeTe_4_ we get an increase in the threshold voltage from 1.2 V to 1.55 V and threshold current from 0.6 µA to 7.2 µA, and more than one order of magnitude decrease in the half bias nonlinearity from 5 × 10^3^ to 10^2^. The slightly asymmetric switching behaviour is due to processing marginalities. For single polarity switching (Supplementary Fig. S1(a)), there is a small variability in the threshold voltage, but clear volatile switching is observed in GeTe_4_ by applying 10 consecutive positive sweeps. For the first route, we obtain an improved thermal stability but a much worse electrical behaviour, which we will discuss later on, in connection with the subthreshold material parameters extraction.

Through the second route, by adding Si, we obtain the desired increase in the crystallization temperature (Fig. 3(a) and Supplementary Fig. S8). For 5% Si, Te crystallization temperature increased to 235 °C and that of GeTe to 285 °C, whereas for 10% Si, Te crystallizes at 280 °C and GeTe at 300 °C. The crystalline phases are stabilized at this Si concentration and adding 15% Si has no additional effect. Even if we were able to push the crystallization temperature to 280 °C, Te crystalline peak disappears below 400 °C, probably due to the melting of the eutectic composition. Figure 3(c) shows that the electrical behaviour changes drastically by the addition of Si. The switching is volatile for 3% silicon, but starting from 5% we have a combination of volatile and non-volatile switching and the non-volatile part increases by increasing Si doping. This transition is clearly observed for 5% Si. If we follow both the median and the average sweeps on the negative polarity (blue and violet curves) there is a clear change from a set to a reset after a number of sweeps, probably necessary for a filament formation. In addition, in support of filamentary-like switching in Si-doped GeTe_6_ devices, we remark a feature (denoted with a circle on the negative polarity in Fig. 3(c) for 7% Si), which suggests the rupture of a filament. Either Si^47^ or Te^48^ filamentary switching, shown recently in CBRAM cells, might explain the non-volatile part, but the formation of a Si filament is more likely since it becomes more pronounced by the addition of Si. Even if the non-volatile part increases after 5% Si doping, we are still able to perform unipolar switching for all tested compositions (Supplementary Fig. S1(b)). For high amounts of Si (e.g. 15% Si) the probability for the device to get stuck in the LRS increases, but the HRS is recovered by applying a negative sweep, which suggests that we are breaking a formed filament. In (Ge_15_Te_85_)_1-x_Si_x_ (1 < × < 6) a memory switching (OMS) effect was observed together with the increase of the switching field as the quantity of Si increases^49^. For Si:GeTe_6_ we are able to delay the crystallization of the material up to 280 °C, and the electrical behaviour is modified. The threshold voltage slightly increases to 1.3 V for 3% Si and then to ~1.7 V, where it stays constant up to 10% Si. The threshold current linearly increases by Si addition to 3.1, 4.3, 5.4, and 7.2 µA, for 3% Si, 5% Si, 7% Si and 10% Si respectively. NL_1/2_ gradually decreases to 100, 50, 10 and 5 for the same amounts of Si doping.

The third and last route is to replace Ge with Si. Si-Te is very similar with Ge-Te but we might expect a higher crystallization temperature since the eutectic point in Si-Te phase diagram for the bulk compound is with 30 °C higher than the eutectic point for Ge-Te, and its melting point is above 400 °C. In PCM, when Ge from Ge_2_Sb_2_Te_5_ is replaced with Si resulting Si_2_Sb_2_Te_5_^50^, the crystallization temperature increases and a lower threshold current is obtained. Recently, it was shown that the simple binary system SiTe^51^ has remarkable electrical properties and the required thermal stability. For the compositions rich in Te, Te starts to crystallize at 110 °C ((100) and (101) hexagonal crystalline peaks are visible in IS-XRD, Supplementary Fig. S9). By increasing the Si amount, the crystallization temperature increases to 200 °C for 30% Si. At this composition the (004) crystalline peak of hexagonal Si_2_Te_3_ starts to become visible in the diffractogram at 360 °C. By further increasing the Si amount, Si_2_Te_3_ remains the only crystalline phase at higher Si concentration (above 50%). Zhang, S. *et al*.^52^ reported for SiTe_6_ and SiTe_4_ crystallization temperatures of 180 °C and 236 °C, respectively. Both amorphous samples were transformed into crystalline Te and Si_2_Te_3_. The discrepancies with our results are probably due to the local compositional non-uniformity, thermal processing induced during the combinatorial deposition and different thickness of the films. In ref. 53, the reported crystallization temperature for Si_2_Te_3_ is 290 °C, which is close to 320 °C, the value we obtained from combinatorial deposition. For SiTe, we obtained a crystallization temperature above 400 °C, whereas Koo, Y. *et al*.^51^ also found that SiTe is stable above this temperature. From the DC bipolar sweeps we can notice, similar to the previous route results, a combination of volatile and non-volatile switching. Threshold switching is present up to 25% Si (i.e. for SiTe_6_, SiTe_4_ and SiTe_3_). Above this concentration (e.g. for SiTe_2_ and SiTe) the electrical characteristics strongly degrade. Unlike ref. 51, we did not obtain a well behaved OTS effect in SiTe. There are several differences between our work and the work from ref. 51. First of all, the thickness of the thin films is different, we have 30 nm as compared with 13 nm in ref. 51 (the threshold voltage is dependent on film thickness *V*_*th*_ ~ *l* ^32^. Secondly, the material of the electrodes has an important influence on the device characteristics^54^ and we used TiN instead of W. This could lead to a higher threshold voltage and a higher threshold current. Finally, regarding the thermal stability, the thermal history influences the properties and microstructure (hence the density of traps) of chalcogenide glasses and thin films^55^. The combinatorial deposition can produce small local compositional non-uniformities which can be viewed as a possibly different process/thermal history. The crystallization temperature also depends on the film thickness^56^. Unipolar switching is only possible for the first three compositions (Supplementary Fig. S1(c)). The threshold voltage increases when moving from SiTe_6_ to SiTe_4_ from 1.45 V to above 2.1 V, then it varies around this value when further increasing the Si amount. The threshold current is low up to SiTe_3_ (3 µA) and then it increases considerably (up to 22 µA for SiTe_2_). Regarding the non-linearity, it is below 5 for all compositions except SiTe_6_, where NL_1/2_ = 100. To conclude, in the Si-Te system, the crystallization of Te starts earlier for Te-rich compositions leading to a low thermal stability. For Si-rich compositions, a higher thermal stability can be achieved, even above BEOL limit, but with a lower electrical performance than the Ge-Te system. The switching parameters for all three routes are summarized in Supplementary Fig. S2.

**Figure 4:**
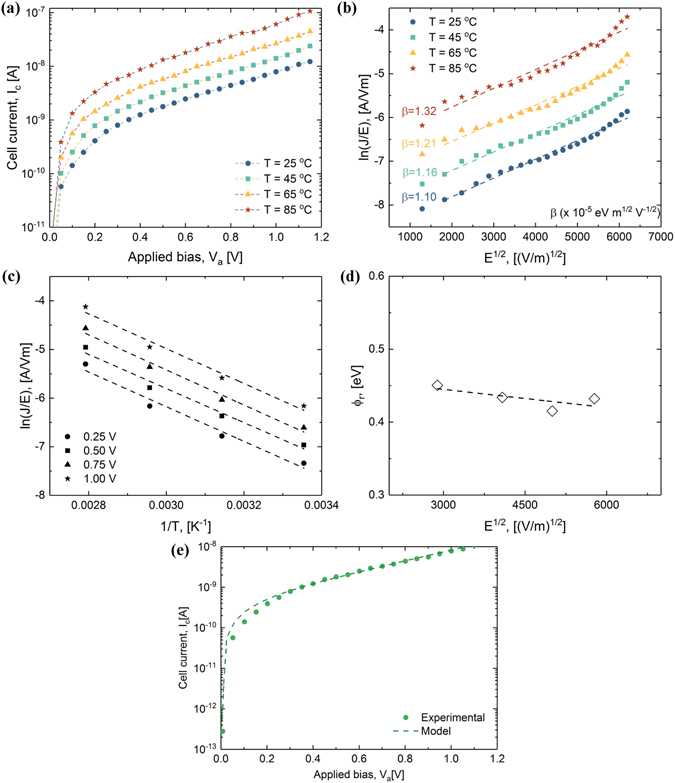
Sub-threshold conduction in GeTe_6_. (**a**): I–V characteristics at four different temperatures, namely 25 °C, 45 °C, 65 °C and 85 °C. (**b**): Poole-Frenkel plot of the sub-threshold current for the temperature ranging from 25 °C to 85 °C. (**c**): Trap depth reduction at various values of the electric field (**d**): Trap depth extraction when no field is applied determined by linear extrapolation (**e**): Modelling of the sub-threshold conduction using Poole-Frenkel model at 25 °C.

The standard Poole-Frenkel mechanism is used to explain the device current dependence on temperature in the sub-threshold regime^57^. This model considers a single trap-energy level, nevertheless assuming the presence of a significant number of acceptor sites^58^. In our work, this analysis is performed on fresh (unoperated) samples, to allow a quantitative comparison of the various films, from electrical response perspective, based on the observed IV dependence on temperature, current-area scaling and reasonable fitting quality and extracted parameter values. The validity of the model/mechanism is not extended or discussed beyond this framework. As shown in literature^30^, the conduction mechanism of operated OTS devices below the threshold remains controversial and its careful investigation is beyond the scope of this work. By applying an electric field the emission probability of a charge carrier from the Coulomb potential of defect state in the band-gap increases. The model can be successfully used to extract the potential depth of these traps. From PF constant $\beta =\sqrt{{q}^{3}/\pi {\varepsilon }_{0}k}$, we can extract the dielectric constant *k* at high frequencies, which can be used as a validation parameter. Figure 4 shows how this model can be applied to GeTe_6_. The current is increasing when rising the temperature from 25 °C to 85 °C (Fig. 4(a)), due to the increase of emission probability. Plotting the logarithm of the current density divided by the field as a function of the square root of the field (Fig. 4(b)), reveals a linear dependence, suggesting that the conduction is controlled by the PF mechanism. Using linear regression we obtain the PF constant (*β* = 1.32) and find the dielectric constant value of *k* = 33. This is an acceptable value if we compare it with the experimental value for amorphous GeTe^59^ (*k* = 23). If the same logarithm is plotted as a function of inverse temperature at different values of the electrical field, we observe that the trap depth reduction increases by increasing the applied voltage and the reduced trap depth can be derived from the slope of each line (Fig. 4(c)). Finally, Fig. 4(d) presents the linear dependence of the reduced trap depth on the applied electric field which shows *ϕ* = 0.47 eV for GeTe_6_, when no voltage is applied. This value of trap depth is higher than the shallow ( ~ 0.25 eV) or deep defect ( ~ 0.4 eV) states in GeTe^60^ or Ge_2_Sb_2_Te_5_^61^. The band-gap that we found for GeTe_6_ by spectroscopic ellipsometry (see Supplementary Fig. S3), namely *E*_*g*_ = 0.82 eV, which is higher than the band-gap of GeTe^62^ (0.45 eV), Ge_2_Sb_2_Te_5_^63^ (0.77 eV) or SiTe_6_^53^ (0.75 eV), is consistent with the higher trap depth found.

The Poole-Frenkel model^34^ fits well the sub-threshold conduction data, with an extracted density of defects *N*_*T*_ = 10.87 × 10^18^ traps/cm^3^. A characteristic attempt-to-escape time (*τ*_0_) of 10^−15^ s^34^ was used for fitting. The value obtained for *E*_*C*_ − *E*_*F*_ = 0.465 eV suggests that the Fermi level *E*_*F*_ is closer to the valence^64^ band rather than being pinned in the middle of the band-gap^65, 66^ as is generally believed. The inter-trap distance *Δz* = 5.3 nm found, somewhat smaller than for Ge_2_Sb_2_Te_5_^67^ (*Δz* = 6.8 nm), is explained by a lower density of defects (2.57 × 10^18^ traps/cm^3^) in GST and is comparable to the inter-trap distance of SiTe^51^ (*Δz* = 5.2 nm). Recently^64^, it was shown that the inter-trap distance has a temperature dependence in addition to the trap depth and density of traps, and the occupation probability of the traps is also important. It has been suggested that the origin of traps responsible for OTS in chalcogenide materials, is mainly related to the non-bonding lone-pair of *p* electrons in the chalcogen atoms^68^. These lone-pair (LP) electrons can create localized states in the band-gap through the interaction with the LP electrons from another atom or with their neighbours^69^. Once created, these defects can be easily charged^70^, e.g. two three-fold coordinated chalcogen atoms can easily form a positively charged three-fold site (C_3_^+^) and a negatively charged one-fold site (C_1_^−^) which will act as donor-like and acceptor-like traps. The right amount of C_1_^−^/C_3_^+^ traps (*N*_*T*_) needs to form/exist and have a certain distribution (*ϕ*) in order for OTS to occur. The interplay between the trap density and trap depth in chalcogenides could be a key for designing OTS materials with the desired electrical characteristics.

**Figure 5:**
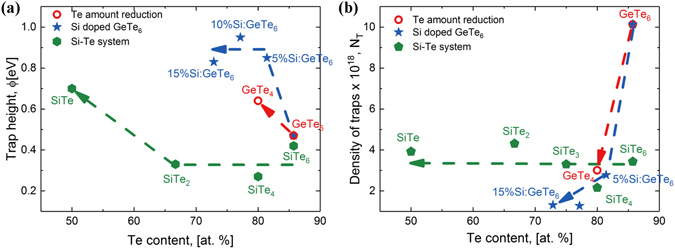
Subthreshold conduction parameters in Ge-Te and Si-Te systems. (**a**): Variation of trap depth extracted using the Poole-Frenkel equation for all the studied materials (**b**): Density of defects as a function of Te amount derived from Poole-Frenkel model.

In a similar manner to that discussed for GeTe_6_, the trap height and the density of traps are extracted for all the studied materials and are summarized in Fig. 5. Through the first two routes, we obtain an increase in *ϕ* to 0.64 eV for GeTe_4_ and to 0.8–0.9 eV for Si:GeTe_6_ (Fig. 5(a)). For the third route, a value of 0.42 eV is obtained for SiTe_6_ and by increasing the Si amount the trap depth remains at 0.3 eV up to 30% Si, then increases to 0.7 eV for SiTe. Regarding the Ge-Te system, both Te reduction and Si doping lead to an increase in the trap depth and a decrease in the density of defects as compared with the starting GeTe_6_ composition. The trap density (Fig. 5(b)) strongly decreases by the first two routes. For the third route it is rather constant. An explanation could be that by reducing the amount of Te, the number of LP electrons will reduce, too, which will lead to the creation of fewer gap states, consistent with our findings. This suggests that both the increase in trap depth and the decrease in the density of defects are responsible for the degradation of the threshold switching in Si:GeTe_6_. Even if the trap depth for SiTe_6_ is comparable with the trap depth for GeTe_6_, the TS effect is not satisfactory because a higher density of traps is needed. A correlation between the increase in the threshold voltage and the increase in the trap depth is observed for the first two routes, whereas the correlation between the decrease in non-linearity and the decrease of the density of defects is evidenced for all three routes.

**Figure 6:**
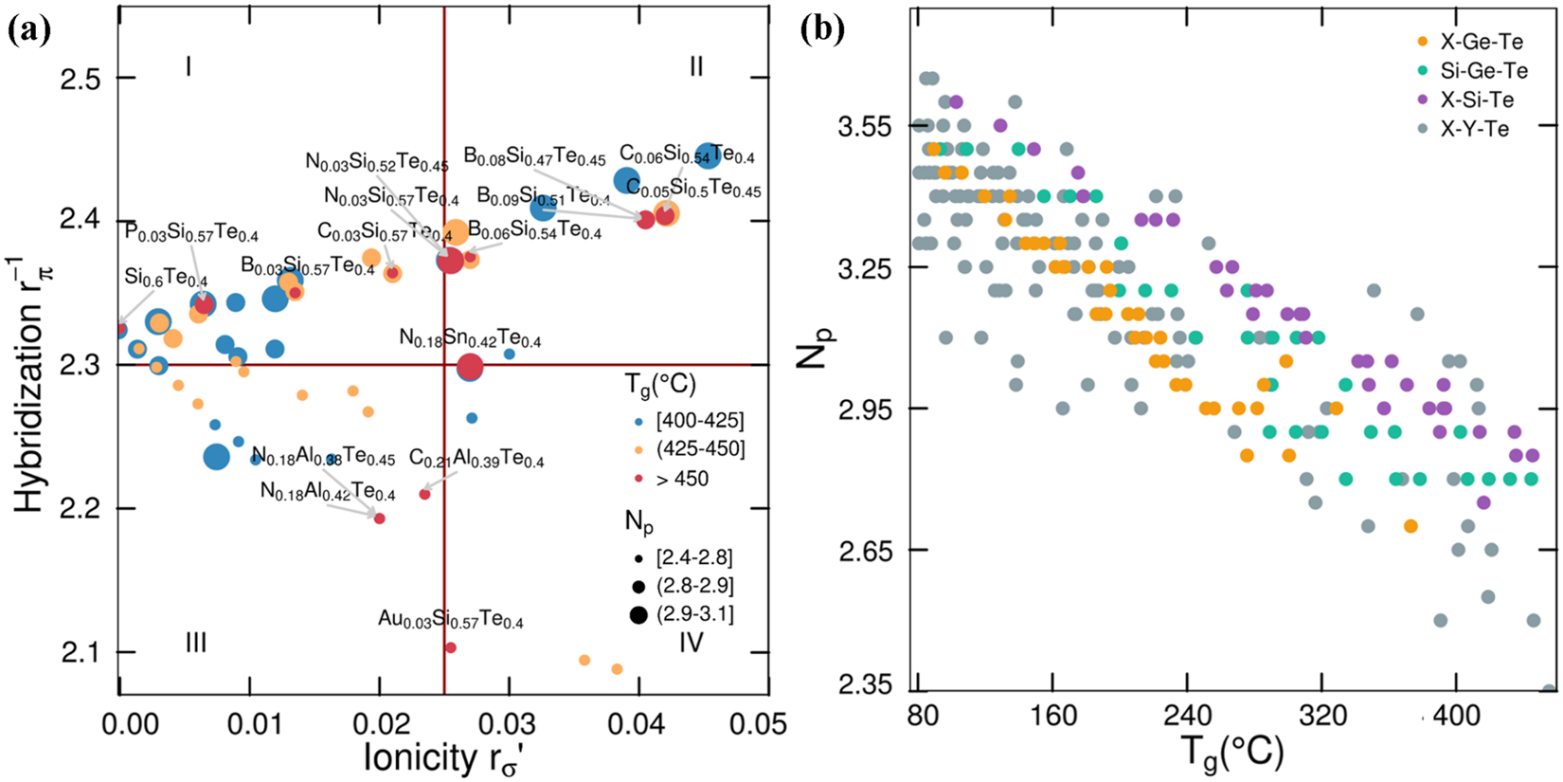
Computed thermal stability of predicted materials exhibiting OTS. (**a**) The glass transition temperature (*T*_*g*_) is used as a monitor of thermal stability. Labels are added to the compositions with *T*_*g*_ > 450 °C. The size of the points is proportional with *N*_*p*_. Zone IV is depopulated and the materials with the highest thermal stability are situated in zones I and II. (**b**) The average number of *p*-electrons as a function of *T*_*g*_. A clear correlation is observed, the average number of *p*-electrons decreases with increasing thermal stability.

As discussed so far, it is difficult to find a binary Te-based chalcogenide material to have the required thermal stability, which could be limited to only several compositions. Therefore, we need to find a way to estimate the thermal stability, especially in ternary compounds. For this, we use the Lankhorst model^46^ which computes the glass transition temperature (*T*_*g*_). The model usually underestimates^71^ *T*_*g*_, but this is not an issue for us because we use it as the lower limit for crystallization, i.e. a worst-case estimate. To validate the method we compare the crystallization temperature (*T*_*c*_) with the glass transition temperature for several compositions (Supplementary Fig. S4(a)) that we prepare and measure. The experimental results are consistent with the computed values. Furthermore, we test the model on additional materials from literature data (Supplementary Fig. S4(b)). Using the Lankhorst model, we compute the thermal stability for possible combinations of *A*_*x*_ *B*_*1-x*_ *Te*_*y*_. Te concentration was varied between 40 and 85, since no OTS material with less than 40% Te or more than 85% was reported in the literature. *A* and *B* are any of the following elements: Cu, Ag, Au, Zn, B, Al, In, C, Si, Ge, Sn, N, P, As and Sb. In addition to the thermal stability, the bond-orbital coordinates and the average number of *p* electrons are also computed. Only the compositions which are located in the area for OTS materials defined in Fig. 1 and with a *T*_*g*_ higher that 400 °C are kept (as shown in Fig. 6(a)). The entire list is given in Supplementary Table S2 from supplementary information. For binary chalcogenides, a limited composition range offers the required thermal stability: Si_0.60_Te_0.40_, Si_0.55_Te_0.45_ and SiTe which has a computed *T*_*g*_ of 373 °C. Even if very recent results, from ref. 51, showed that materials with similar binary compositions exhibit good OTS switching, it is likely that the narrow composition range will make it difficult, if not hardly possible, to tune the various electrical parameters relevant for 1S1R operation, while remaining self-confined in the binary Si-Te space. Eye-opening is the fact that materials compositions with a higher thermal stability have a maximum *N*_*p*_ of 3.15 (Fig. 6(b)). We can observe a slower decrease of *N*_*p*_ for X-Si-Te materials as-compared with X-Ge-Te, which confirms that Si containing Te-based chalcogenide materials can reach higher thermal stabilities, as also shown by Fig. 3(a). It seems that there is a trade-off between electrical performance and thermal stability.

In order to test the predictive power of the model, in addition to the Si-Te system, which already confirms a crystallization temperature above BEOL integration for Si amount > 50%, we prepare and assess the thermal stability of several materials from the C-Si-Te and Sn-Si-Te systems. The IS-XRD spectra of these compositions confirm the predicted thermal stability (Supplementary Fig. S5).

## Conclusion

To summarize, we propose a new pathway for OTS materials design and discovery that brings a more systematic search approach based on materials properties. The investigation of GeTe_6_ showed that this composition has excellent electrical properties but fails to meet the thermal stability requirements. All the routes we followed to increase the thermal stability succeeded, but at the cost of electrical switching degradation. The presence of Si introduces a combination of volatile and non-volatile switching that could be related to the formation of a filament inside the material. The poor electrical behaviour is also related to the trap density decrease caused by the reduction of chalcogen atoms and therefore of the LP electrons of the compound.

An optimum solution combining thermal stability and threshold switching is yet to be found, possibly beyond the binary chalcogenide materials space. We highlighted promising candidates for thermally stable ternary OTS materials. A trade-off is to be made between thermal stability and strong/well-behaved OTS. These guidelines may serve for material selection in selector applications.

## Methods

### Bond-orbital coordinates

The bond orbital coordinates were first defined by St. John and Bloch^72^ based on the orbital radii of Simons and Bloch^73^ and they applied these coordinates to predict the crystal structures of solids. With an accuracy of 90–95% they were able to separate the most stable structures. Littlewood^74^ slightly modified these two coordinates in order to obtain 100% separation between the three crystal structures (cubic, rhombohedral and orthorhombic) and he named them ‘ionicity’ (${r}_{\sigma }^{\text{'}}$) and ‘covalency’ (${r}_{\pi }^{-1}$). These two coordinates were later extended by Lencer *et al*.^36^ to multinary compounds. In the present study, we use the ionicity and covalency defined in eqs. (1) and (2):

1 $${r}_{\sigma }^{\text{'}}=|(\frac{{\sum }_{i}{n}_{i}{r}_{p,i}}{{\sum }_{i}{n}_{i}})-(\frac{{\sum }_{j}{n}_{j}{r}_{p,j}}{{\sum }_{j}{n}_{j}})|$$

2 $${r}_{\pi }^{-1}={[(\frac{{\sum }_{i}{n}_{i}({r}_{p,i}-{r}_{s,i})}{{\sum }_{i}{n}_{i}})+(\frac{{\sum }_{j}{n}_{j}({r}_{p,j}-{r}_{s,j})}{{\sum }_{j}{n}_{j}})]}^{-1}$$

where *r*_*s*_ and *r*_*p*_ are the *s* and *p* orbital radii and *n*_*i*_ the number of atoms of species *i* per formula unit. Increasing hybridization leads to increased distortions and to stronger covalent bond formation, while increasing ionicity leads to localized charge at the ions cores which will increase the probability of ionic bond formation. The average number of *p*-electrons (*N*_*p*_) is computed using eq. (3), adapted from ref. 75:

3 $${N}_{p}=\frac{{\sum }_{i}{n}_{i}{p}_{i}}{{\sum }_{i}{n}_{i}}$$

with *n*_*i*_ the number of atoms per species *i* per formula unit and *p*_*i*_ the number of *p*-electrons per species *i*.

### Experimental

The samples are deposited by DC-magnetron sputtering from Ge, Si and Te sputter targets, using a commercial Balzers BAS 450 deposition tool. Substrates are mounted on a rotating carousel in the deposition chamber with a base pressure of 5 × 10^−7^ mbar. Passing subsequently in front of each sputter target, results in intermixed Ge - Te and Si - Te layers. Materials properties are investigated on n-doped Si(100) substrates, covered with 10 nm SiO_2_, with the films deposited between TiN layers, in order to resemble as much as possible the device stack. For the Si-Te system we used combinatorial deposition on a 6″ wafer.

The Si-Te composition of the films is determined by energy-dispersive X-ray spectroscopy (EDX), using a FEI Quanta 200F FEG scanning electron microscope (SEM) equipped with a Genesis 4000 EDX detector, after calibration with RBS. X-ray fluorescence spectroscopy (XRF) is used to determine the Ge-Te compositions, after calibration of the system by RBS.

The thermal stability of the material is investigated by means of *in situ* X-ray Diffraction (IS-XRD). IS-XRD allows to characterize the phase formation as a function of temperature and hence gives information about phase transformations. A customized setup consisting of a heating chamber mounted in a Bruker D8 Discover XRD system is used. The samples are subjected to a constant heating rate of maximum 0.5 °C/s under an inert He atmosphere, while a diffraction pattern is recorded every 4 s over a fixed 2θ window.

The preparation process of cross-bar devices for electrical characterization is sketched in Supplementary Fig. S6. We start with a Si wafer, then a TiN bottom electrode (BE) is patterned by photolithography, etched and planarized by chemical mechanical polishing. We apply the sequence of steps 1 to 5, as shown in Supplementary Fig. S6. First, the wafer is coated with a photoresist, followed by e-beam exposure using a Vistec VB6 electron beam lithography tool. After the exposure, the photoresist is developed and the chalcogenide material (CM) together with the top electrode (TE) are deposited. Further, after the deposition of the full stack, lift-off is performed. The device is defined as the overlapping region between the bottom and top electrode lines, whereas the device size is given by the width of the lines. The usual thickness of the CM is 30 nm and the thickness of the TiN TE is 20 nm.

Electrical assessment of the devices is performed using a custom built setup, where Keithley K2602A units are used as SMU’s. Double DC sweeps up to ± 5 V are applied and the response in current of the devices is measured. A compliance current (*I*_*cc*_) of maximum 100 µA is used in order to avoid the degradation of the devices.

### Subthreshold conduction mechanisms

The threshold switching mechanism in amorphous chalcogenides is governed by the traps in the bandgap. Poole-Frenkel^33^ model shows how the electron trap depth is reduced by applying an electric field. The dependence of the current density (*J*) on the electric field (*E*) is given in eq. (4), where *q* is the elementary charge, *β* the PF constant and *k*_*B*_ the Boltzmann constant.

4 $$J=CE{e}^{\frac{\beta \sqrt{E}-q\varphi }{{k}_{B}T}}$$

Since the constant *C* is not known, we need data from several temperatures in order to exclude it. The logarithm of the current density divided by the field has a linear dependence on the inverse temperature at different values of the electric field, as follows:

5 $$\mathrm{ln}(\frac{J}{E})=\,\mathrm{ln}(C)-\frac{\beta \sqrt{E}-q\varphi }{{k}_{B}T}\cdot \frac{1}{T}$$

The slope of each line is proportional to the reduced trap depth at that specific electric field. The intercept of the reduced trap depth with the *y* axis at zero field represents the trap depth (*ϕ*). Temperature range or thickness dependence of the trap depth is neglected^76^.

Poole-Frenkel^34^ model considers an amorphous semiconductor with an uniform distribution of traps in the band-gap. The model takes into account the interaction between traps, using the inter-trap distance parameter *Δz*, and the current *I*, which depends on the applied electrical field in the following form:

6 $$I=2qA{N}_{T}\frac{{\rm{\Delta }}z}{{\tau }_{0}}e\frac{-({E}_{C}-{E}_{F})}{{k}_{B}T}\,\sinh (\frac{qE}{{k}_{B}T}\cdot \frac{{\rm{\Delta }}z}{2})$$

where *A* is the area of the contact, *N*_*T*_ the density of traps in the band-gap, *τ*_0_ the characteristic attempt-to-escape time for the trapped electron and *E*_*C*_ *– E*_*F*_ the distance between the Fermi level and the conduction band. Threshold switching is considered to occur through the filling of traps near the conduction band edge at high electric field. A parameter extraction method based on this model has been developed and used to extract the density of traps in the band-gap, on as-processed (pristine) samples.

### Glass transition temperature estimation

Glass transition temperature is the temperature where a glass suffers a transition from a rigid to a soft ‘polymer-like’ material. Since some of the bonds between elements are broken in order for the crystallization to take place, the transition can be measured as an endothermic step in differential scanning calorimetry (DSC). At normal heating rates, amorphous materials crystallize above the glass transition temperature^71^. So, this temperature can be seen as a lower limit for crystallization. Lankhorst^46^ developed a model based on bond enthalpies for the estimation of *T*_*g*_ for glasses predominantly covalently bonded. Depending on the composition of the material, we need to have a representation of the bonds in that material. We begin with the coordination number of each element and start to form bonds, first heteronuclear and then homonuclear in sequence of decreasing bond enthalpy. The process stops when the available formal valences of the atoms are saturated and the total number of bonds divided by the number of atoms equals half of the average coordination number. The enthalpy of atomisation (*H*_*a*_) is calculated by summing all individual bond enthalpies in the structure. Using the empirical relation from eq. (7) derived from a linear regression model, we can compute the glass transition temperature (in *K*):

7 $${T}_{g}=3.44\cdot {H}_{a}-480$$

where 3.44 is the slope in *K/(kJ/mol)* units and 480 is the intercept in *K*.

## Electronic supplementary material

Supplementary Information
