# Supplementary material for: Te-based chalcogenide materials for selector applications
Source: Sci Rep. 2017 Aug 14;7:8103. doi: 10.1038/s41598-017-08251-z (PMC5556072; doi:10.1038/s41598-017-08251-z)
Supplement: Supplementary file 1 — Supplementary Information [file 41598_2017_8251_MOESM1_ESM.pdf]

Te-based chalcogenide materials for selector applications

A. Velea[a, c, *)], K. Opsomer[a], W. Devulder[a], J. Dumortier[b], J. Fan[a], C. Detavernier[b], M. Jurczak[a], B. Govoreanu[a]

[a] *Imec, Kapeldreef 75, 3001 Heverlee, Belgium*
[b] *Ghent University, dept. Solid State Sciences, Krijgslaan 281 (S1), 9000 Ghent, Belgium*
[*)] *also with KU Leuven, Arenbergpark 10, B-3001 Leuven, Belgium*
[c] *National Institute of Materials Physics, Atomistilor 405A, P.O. Box M.G. 7, Magurele, 077125 Ilfov, Romania*

The list of materials and their associated properties used to build the OTS materials map are given in Table ST1. These points cluster and define an area on the *Ionicity = f(Hybridization)* plot that has a high probability to host materials exhibiting OTS. This map is used to predict new selector materials by computing different compositions that lie in the same area.

Table ST1. Materials used to build the OTS map.

Composition	Ionicity	Hybridization	Np
$Ge_{0.5}Te_{0.5}$	0.03	2.08333	3.0
$Si_{32.7}As_{14.3}Te_{53.0}$	0.01826	2.35896	3.203
$Si_{21.1}As_{38.2}Te_{40.7}$	0.03857	2.39726	3.196
$Si_{11.8}As_{24.1}Te_{64.1}$	0.04065	2.40124	3.523
$Si_{6.9}As_{17.9}Te_{75.2}$	0.04333	2.40642	3.683
$Si_{10}As_{30}Se_{10}Te_{50}$	0.02333	2.43902	3.5
$Ge_{10}Si_{30}As_{12}Te_{48}$	0.00933	2.29475	3.08
$Ge_{7}Si_{18}As_{35}Te_{40}$	0.0314	2.35746	3.15
$Ge_{7}Si_{17}As_{36}P_{1}Te_{39}$	0.0341	2.36434	3.15
$Ge_{7.9}Si_{1.7}As_{21.4}Te_{69.0}$	0.03377	2.33135	3.594
$Ge_{8.4}Si_{2.2}As_{22.4}Te_{67.0}$	0.03309	2.33018	3.564
$Ge_{9.8}Si_{1.8}As_{23.9}Te_{64.5}$	0.03211	2.32375	3.529
$Ge_{8.3}Si_{2.0}As_{21.7}Te_{68.0}$	0.03291	2.3288	3.577
$Ge_{9.1}Si_{2.1}As_{24.3}Te_{64.5}$	0.03338	2.33031	3.533
$Ge_{9.6}Si_{2.0}As_{24.4}Te_{64.0}$	0.03267	2.32678	3.524
$Ge_{8.7}Si_{1.9}As_{22.4}Te_{67.0}$	0.03282	2.32771	3.564
$Ge_{9.7}Si_{1.9}As_{24.4}Te_{64.0}$	0.03258	2.32603	3.524
$Ge_{7.4}Si_{2.1}As_{22.0}Te_{68.5}$	0.03486	2.33766	3.59
$Ge_{8.9}Si_{2.0}As_{24.1}Te_{65.0}$	0.03369	2.33131	3.541

Single polarity switching sweeps for the three studied routes are shown in Fig. S1. The threshold switching is observed in all graphs.

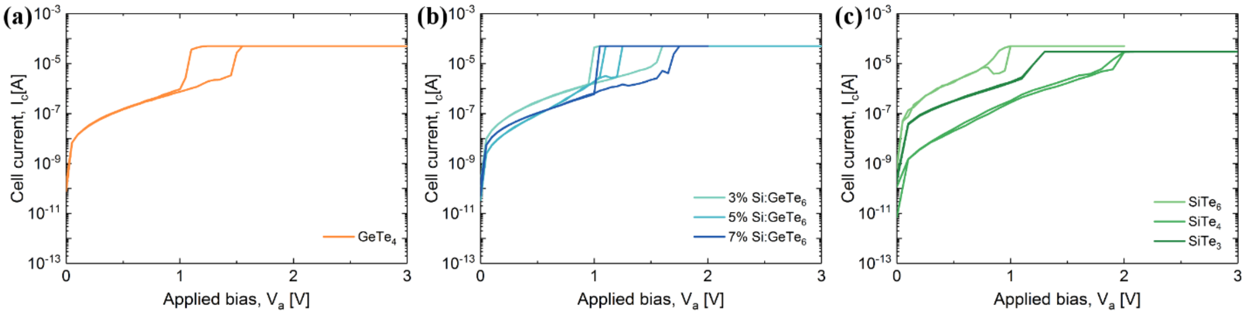

Figure S1. Positive polarity switching. (**a**): $GeTe_4$ (**b**): Si doped $GeTe_6$ (**c**) Si-Te system. Each curve is the median of 10 sweeps. 30 µA and 50 µA compliance currents were used.

A summary of switching parameters and electrical performance for the studied materials is given in Fig. S2. All three routes conduct to a degradation of the electrical characteristics.

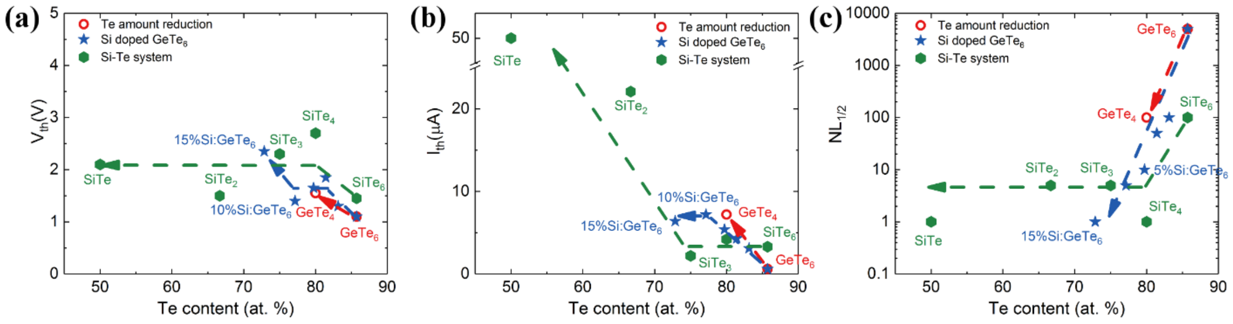

Figure S2. Switching parameters for the followed routes. (**a**): Threshold voltage (**b**): Threshold current and (**c**) Half bias non-linearity

A Woollam Vertical-Variable Angle Spectroscopic Ellipsometer (V-VASE) was used for optical characterization of the $GeTe_6$ film. Measurements were performed from UV to near infrared (250–1700 nm), at fixed angle of incidence (AOI) of 70°. Tauc-Lorentz model[1] ($\varepsilon_2(E) = \frac{(E - E_g)^2}{E} \cdot \frac{AE_0\Gamma}{\left[(E - E_0^2)^2 + \Gamma^2 E^2\right]}$) was used for the estimation of other optical parameters of the film. This model was successfully used earlier on GST materials[2]. The parameters of the model are: E_g - the optical band gap, E_0 - the resonance energy (critical energy), A - the amplitude and Γ - the broadening parameter of the oscillator. As presented in Fig. S3(a) and (b) the model fits well the experimental data (MSE = 2.61). The values obtained for the fitting parameters are: A = 121.72 ± 1.56, E_0 = 2.369 ± 0.009 eV, E_g = 0.817 ± 0.004 and Γ = 3.963 ± 0.023. The refractive index and extinction coefficient are shown in Fig. S3(c). The absorption coefficient ($\alpha = \frac{4\pi k}{\lambda}$) from Fig. S3(d), which is between 10^5 and 10^6 cm^{-1}, suggests that we have a direct band-gap[3] in $GeTe_6$.

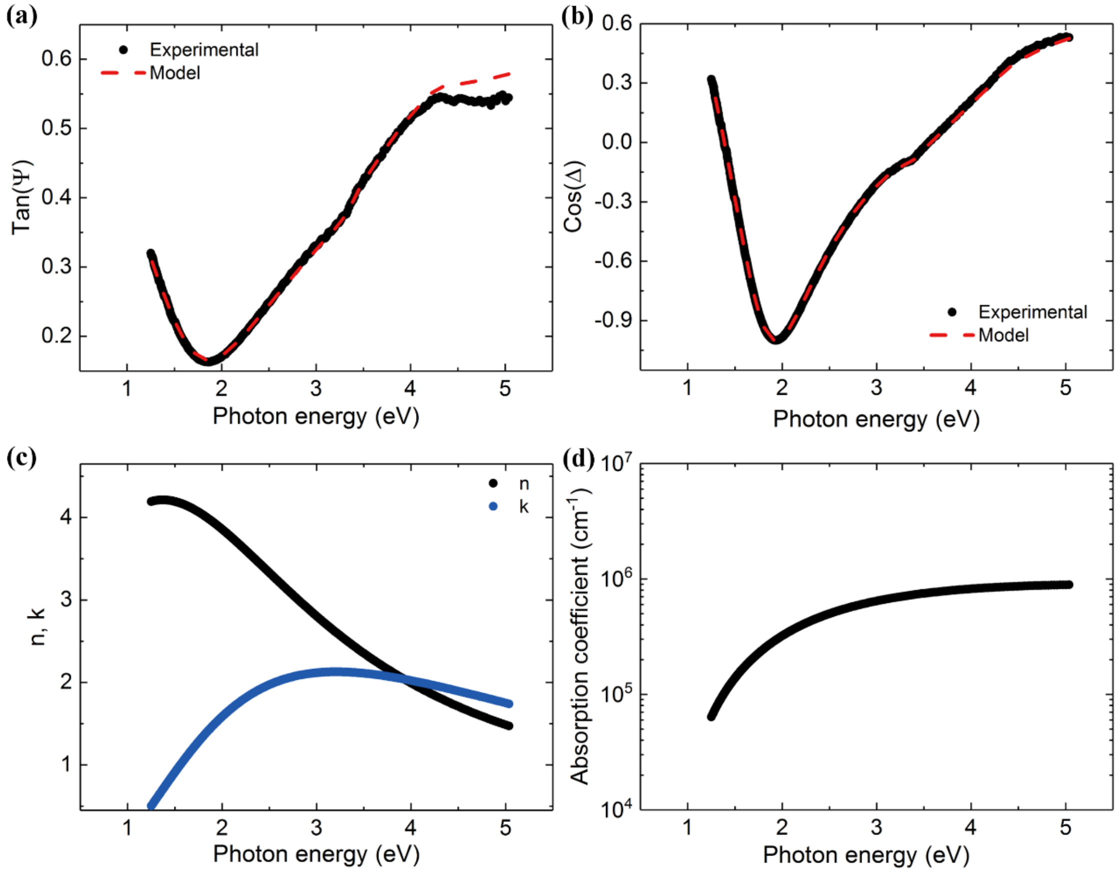

Figure S3. Spectroscopic ellipsometry results for $GeTe_6$. (**a**): Amplitude ratio upon reflection (*tan(Ψ)*) (**b**): Phase shift (*cos(Δ)*); (**c**): Refractive index (*n*) and extinction coefficient (*k*); (**d**): Absorption coefficient (*α*).

As stated in the main text, the glass transition temperature can be used as a lower limit for the crystallization temperature. Fig. S4(a) shows the theoretical glass transition temperature as a function of the experimental crystallization temperature, measured by IS-XRD for several compositions that we prepared in order to test this assumption. For all the compositions from the systems C-Ge-Te, Si-Ge-Te and N-Ge-Te, this assumption holds (i.e T_g^{sim} is always below T_c^{exp}). In addition to our measurements, we explored a series of compositions from previously published data for Ge-As-Te[4] and Si-As-Te[5] systems and compared the experimental and the computed glass transition temperatures (Fig. S4(b)). The model usually underestimates T_g, but the values are acceptable. We can conclude that the computed glass transition temperature is a reliable lower limit for the crystallization temperature.

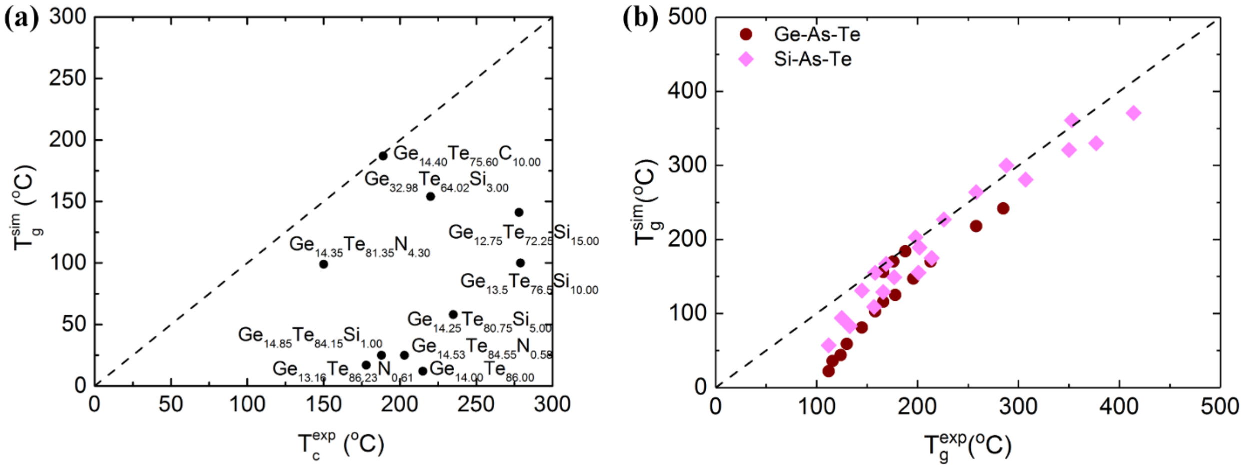

Figure S4. Validation of the Lankhorst method. (**a**): Relation between experimental crystallization temperature (T_c^{exp}) and computed glass transition temperature (T_g^{sim}). All values are situated below the diagonal indicating that T_g^{sim} can be considered the lower limit of T_c^{exp} (**b**): Comparison of experimental and computed T_g in Te-based chalcogenide materials. A good correlation is observed, although the model tends to underestimate T_g.

Using the new map of OTS materials, we predicted a list of materials that should have both the expected thermal stability and OTS property, and are listed in Table ST2.

Table ST2. Predicted compositions with glass transition temperature above 400°C.

Composition	T_g (°C)	N_p	Composition	T_g (°C)	N_p
$Si_{0.60}Te_{0.40}$	457	2.8			
$Si_{0.55}Te_{0.45}$	415	2.9			
$C_{0.03}Si_{0.57}Te_{0.40}$	489	2.8			
$C_{0.0275}Si_{0.5225}Te_{0.45}$	444	2.9			
$Ge_{0.03}Si_{0.57}Te_{0.40}$	450	2.8	$C_{0.06}Si_{0.54}Te_{0.40}$	521	2.8
$Ge_{0.0275}Si_{0.5225}Te_{0.45}$	408	2.9	$C_{0.055}Si_{0.495}Te_{0.45}$	473	2.9
$B_{0.03}Si_{0.57}Te_{0.40}$	476	2.77	$C_{0.05}Si_{0.45}Te_{0.50}$	426	3.0
$B_{0.0275}Si_{0.5225}Te_{0.45}$	432	2.8725	$C_{0.21}Sn_{0.39}Te_{0.40}$	413	2.8
$P_{0.03}Si_{0.57}Te_{0.40}$	451	2.83	$B_{0.06}Si_{0.54}Te_{0.40}$	494	2.74
$P_{0.0275}Si_{0.5225}Te_{0.45}$	409	2.9275	$B_{0.055}Si_{0.495}Te_{0.45}$	449	2.845
$P_{0.06}Si_{0.54}Te_{0.40}$	444	2.86	$B_{0.09}Si_{0.51}Te_{0.40}$	512	2.71
$P_{0.055}Si_{0.495}Te_{0.45}$	403	2.955	$B_{0.0825}Si_{0.4675}Te_{0.45}$	465	2.8175
$P_{0.09}Si_{0.51}Te_{0.40}$	437	2.89	$N_{0.03}Si_{0.57}Te_{0.40}$	499	2.83
$Sn_{0.03}Si_{0.57}Te_{0.40}$	442	2.8	$N_{0.0275}Si_{0.5225}Te_{0.45}$	453	2.9275
$Sn_{0.0275}Si_{0.5225}Te_{0.45}$	401	2.9	$N_{0.025}Si_{0.475}Te_{0.50}$	407	3.025
$Sb_{0.03}Si_{0.57}Te_{0.40}$	439	2.83	$P_{0.12}Si_{0.48}Te_{0.40}$	430	2.92
$Sb_{0.06}Si_{0.54}Te_{0.40}$	420	2.86	$P_{0.15}Si_{0.45}Te_{0.40}$	423	2.95
$Sb_{0.09}Si_{0.51}Te_{0.40}$	401	2.89	$P_{0.18}Si_{0.42}Te_{0.40}$	417	2.98
$As_{0.03}Si_{0.57}Te_{0.40}$	444	2.83	$P_{0.21}Si_{0.39}Te_{0.40}$	410	3.01
$As_{0.0275}Si_{0.5225}Te_{0.45}$	403	2.9275			
$As_{0.06}Si_{0.54}Te_{0.40}$	431	2.86			
$As_{0.09}Si_{0.51}Te_{0.40}$	418	2.89			
$As_{0.12}Si_{0.48}Te_{0.40}$	405	2.92			
$C_{0.21}Al_{0.39}Te_{0.40}$	790	2.41			
$Al_{0.03}Si_{0.57}Te_{0.40}$	448	2.77			
$Al_{0.06}Si_{0.54}Te_{0.40}$	438	2.74			
$Zn_{0.03}Si_{0.57}Te_{0.40}$	424	2.74			
$In_{0.03}Si_{0.57}Te_{0.40}$	433	2.77			
$Sn_{0.06}Si_{0.54}Te_{0.40}$	427	2.8	$Ag_{0.03}Si_{0.57}Te_{0.40}$	432	2.74
$Si_{0.39}Ge_{0.21}Te_{0.40}$	404	2.8	$Au_{0.03}Si_{0.57}Te_{0.40}$	453	2.74
$Si_{0.42}Ge_{0.18}Te_{0.40}$	412	2.8	$N_{0.18}Sn_{0.42}Te_{0.40}$	466	2.98
$Si_{0.45}Ge_{0.15}Te_{0.40}$	419	2.8	$N_{0.165}Sn_{0.385}Te_{0.45}$	422	3.065
$Si_{0.48}Ge_{0.12}Te_{0.40}$	427	2.8	$Cu_{0.03}Si_{0.57}Te_{0.40}$	438	2.74
$Si_{0.51}Ge_{0.09}Te_{0.40}$	435	2.8	$Sn_{0.09}Si_{0.51}Te_{0.40}$	413	2.8
$Si_{0.54}Ge_{0.06}Te_{0.40}$	442	2.8			
$Si_{0.495}Ge_{0.055}Te_{0.45}$	401	2.9			
$N_{0.15}Sn_{0.45}Te_{0.40}$	413	2.95			
$N_{0.18}Al_{0.42}Te_{0.40}$	718	2.56			
$N_{0.165}Al_{0.385}Te_{0.45}$	652	2.68			

Next, we give two examples, for $Si_{0.45}Ge_{0.15}Te_{0.40}$ and $In_{0.05}Si_{0.25}Te_{0.70}$, showing how the glass transition temperature is computed using the Lankhorst model[7]. Homonuclear bond enthalpies (H_{AA} *(kJ/mol)*), Pauling electronegativities (*S*) and average number of valence electrons (*N*), for the elements in the selected compositions, are: H_{GeGe} = 186 *kJ/mol*, S_{Ge} = 2.01, N_{Ge} = 4, H_{TeTe} = 197 *kJ/mol*, S_{Te} = 2.1, N_{Te} = *6*, H_{SiSi} = 225 *kJ/mol*, S_{Si} = 1.9, N_{Si} = 4 and H_{InIn} = 50 *kJ/mol*, S_{In}= 1.78, N_{In} = 3.

The average coordination number $<m>/2$, computed using the generalized $8 - <N>$ rule, is 1.6 and 1.325 for $Si_{0.45}Ge_{0.15}Te_{0.40}$ and $In_{0.05}Si_{0.25}Te_{0.70}$, respectively. The model is applicable only in case N ≥ 4. Therefore, in some cases, formal electron transfer takes place from one atom to another. The second composition was selected to emphasize this charge transfer. So, for $In_{0.05}Si_{0.25}Te_{0.70}$, as a consequence of formal electron transfer from Te to In, all In atoms will form four bonds (0.15) and 0.05 Te atoms will form three bonds in order to satisfy the total number of bonds in the structure.

The heteronuclear bond enthalpies (H_{AB}) are computed using the Pauling equation: $H_{AB} = \frac{(H_{AA} + H_{BB})}{2} + 96.14 \cdot (S_A - S_B)^2$. So, we obtain H_{SiTe} = 215 *kJ/mol*, H_{SiGe} = 206 *kJ/mol* and H_{InTe} = 133 *kJ/mol*.

The bonds are then formed in the order of decreasing bond enthalpies, heteronuclear bonds being preferred to homonuclear bonds, as shown in Table ST3.

Table ST3. Calculated glass transition temperature and bond enthalpies for $Si_{0.45}Ge_{0.15}Te_{0.40}$ and $In_{0.05}Si_{0.25}Te_{0.70}$. For each bond, the number of bonds and the enthalpy these bonds are contributing to the enthalpy of atomisation, are shown. The enthalpies are given in *kJ/mo*l, and T_g is in *°C*.

Composition	n_{SiTe}	$n_{SiTe}H_{SiTe}$	n_{SiGe}	$n_{SiGe}H_{SiGe}$	n_{InTe}*	$n_{InTe}H_{InTe}$	n_{SiSi}	$n_{SiSi}H_{SiSi}$	n_{TeTe}	$n_{TeTe}H_{TeTe}$	H_a	T_g
$Si_{0.45}Ge_{0.15}Te_{0.40}$	0.8	172	0.6	124	-	-	0.2	45	-	-	341	419
$In_{0.05}Si_{0.25}Te_{0.70}$	1.0	215	-	-	0.2	27	-	-	0.125	25	267	165

*Bond with formal charge transfer

Finally, the enthalpy of atomisation, H_a, is the summation over the enthalpy contribution from each type of bond. The glass transition temperature is then computed using eq. (7) from the manuscript.

The first two compositions from table ST2 ($Si_{0.60}Te_{0.40}$ and $Si_{0.55}Te_{0.45}$), proved experimentally to have the expected thermal stability (Fig. S5(a) and (b)), but showed a poor OTS effect. In addition, we prepared two other compositions from the system C-Si-Te, namely $C_{0.0275}Si_{0.5225}Te_{0.45}$ and $C_{0.05}Si_{0.45}Te_{0.50}$ (Fig. S5(c) and (d)), and another composition from the Sn-Si-Te system ($Sn_{0.06}Si_{0.54}Te_{0.40}$ - Fig. S5(e)). Their thermal stability was tested by IS-XRD

(Fig. S5). Both of them have the crystallization temperatures above the predicted glass transition temperatures of 444 °C, 473 °C and 427 °C, respectively. Only the peaks of crystalline Si_2Te_3 are visible for the first four compositions. Additional peaks due to the TiN cap or Si substrates are recognizable in the diffractograms.

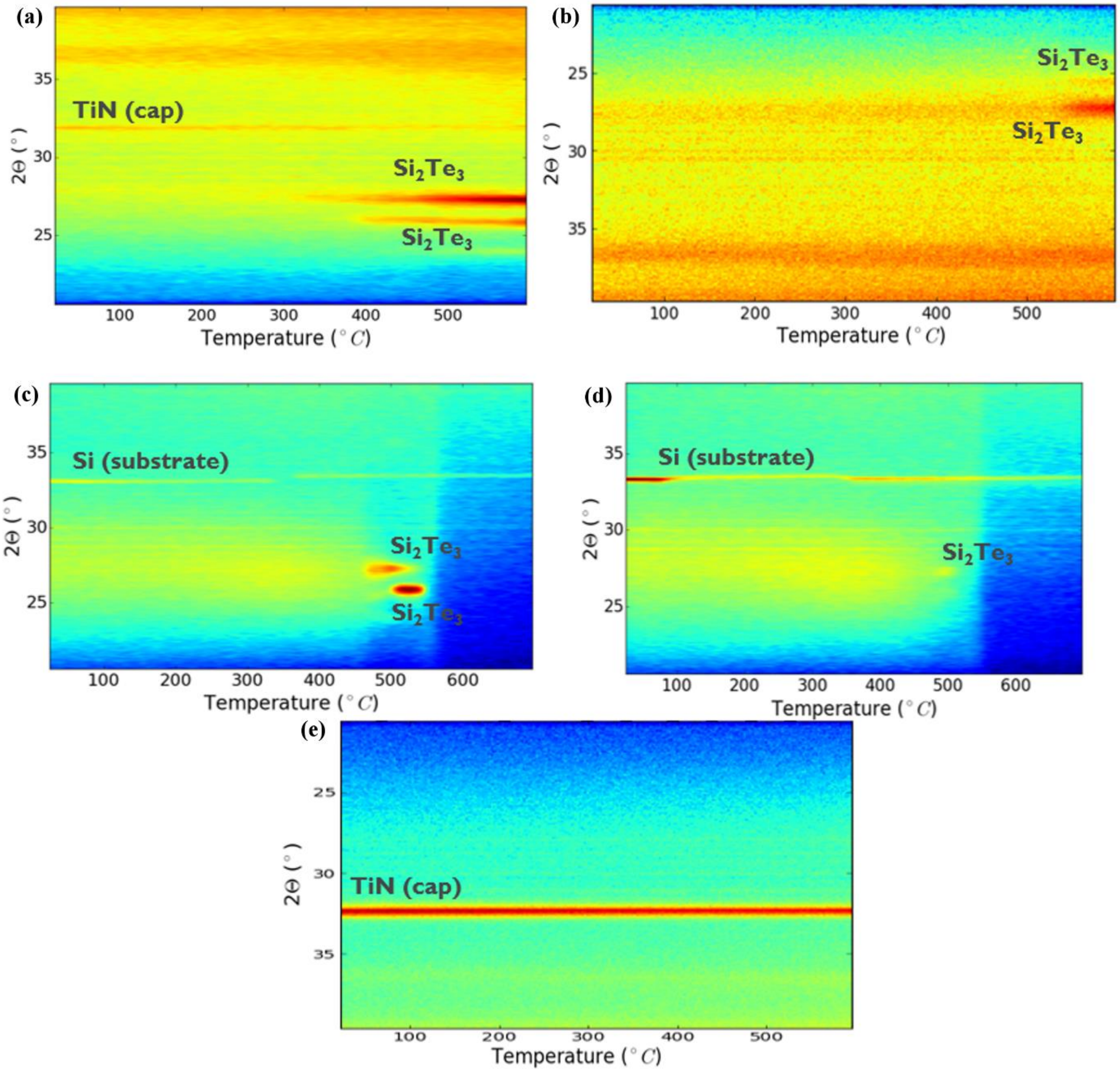

Figure S5. IS-XRD spectra of several predicted compositions. (**a**): $Si_{0.55}Te_{0.45}$ (**b**): $Si_{0.60}Te_{0.40}$ (**c**): $C_{0.0275}Si_{0.5225}Te_{0.45}$ (**d**): $C_{0.05}Si_{0.45}Te_{0.50}$ (**e**): $Sn_{0.06}Si_{0.54}Te_{0.40}$

Device fabrication process procedure is shown in Fig. S6. The devices are fabricated using an e-beam & lift-off process, on substrates pre-processed in a 300 mm process. The substrate pre-processing involves definition and patterning of a TiN bottom electrode (BE) on a thick oxide substrate, deposited on 300 mm Si wafers. The BE patterning is followed by oxide deposition and chemical-mechanical polishing (CMP), stopping on TiN BE, so as to end up with a planarized BE top-surface. The wafers processed up to this point follow a process similar to that of Ref. 6, and serve as substrate for the subsequent lab processing (starting at cartoon 1), where the devices are processed to define the top electrode stack (i.e. TE contact and the chalcogenide film). The final device is a crossbar, where a chalcogenide film is sandwiched between two TiN crossing electrodes. Devices with dimension from 10 μm, down to 55 nm are patterned using this flow.

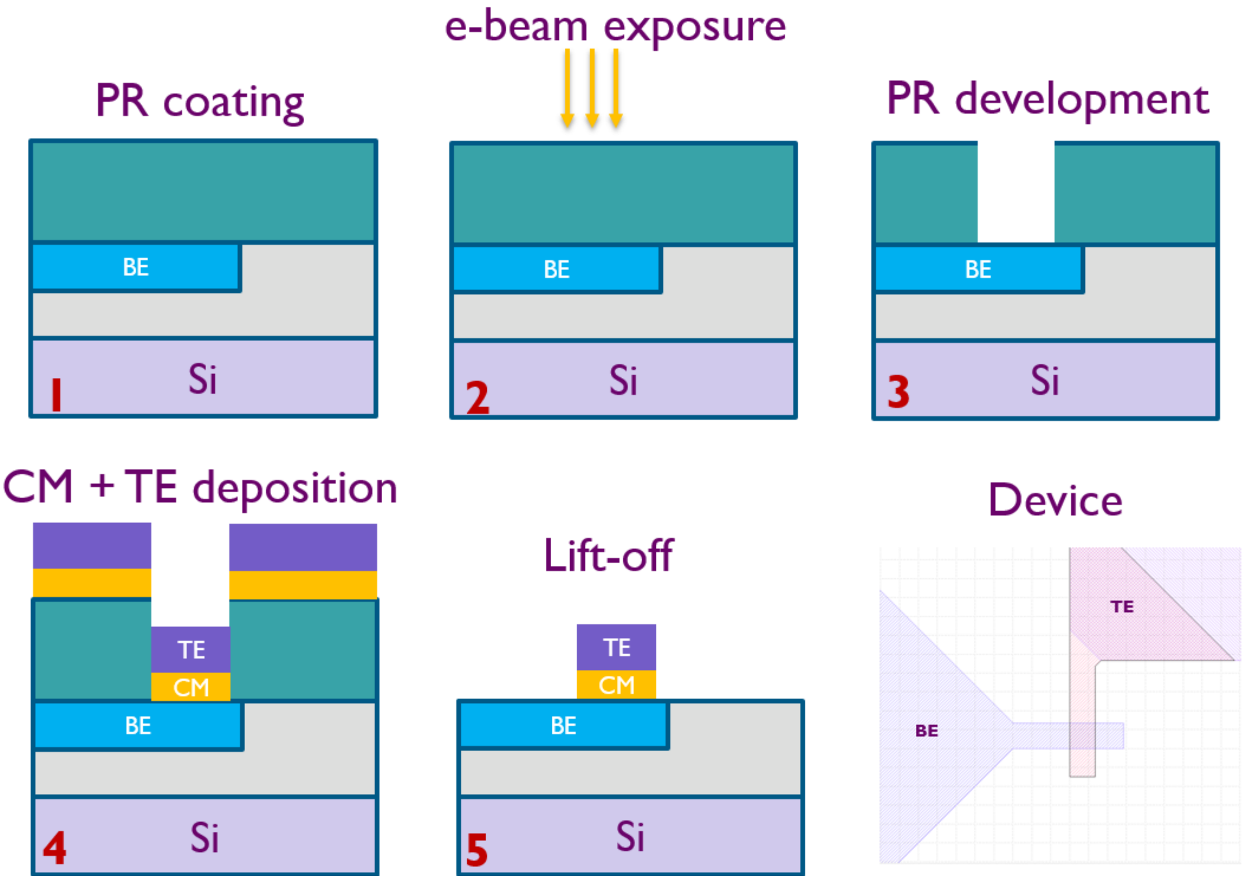

Figure S6. Schematic sample preparation flow (transversal view): 1) Photoresist coating; **2**) e-beam exposure; **3**) Photoresist development; **4**) PVD deposition of binary chalcogenide material (CM) and top electrode (TE); **5**) Lift-off process. Finally a top-view sketch of a device is shown.

IS-XRD data for all three routes of thermal stability increase are shown in Figs. S7, S8 and S9. All the routes followed to increase the thermal stability succeeded, but at the cost of electrical switching degradation.

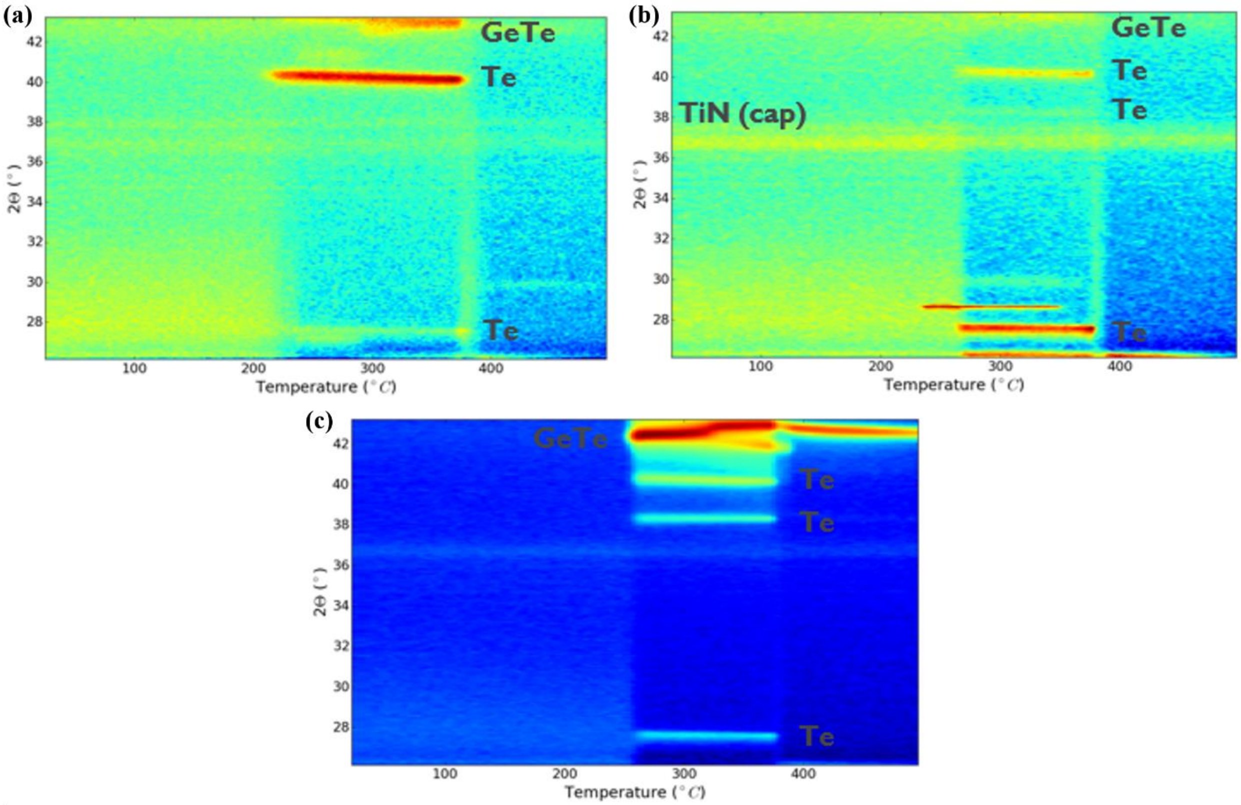

Figure S7. IS-XRD spectra for the compositions used in the first route (Te amount reduction). (**a**): $GeTe_6$ (**b**): $GeTe_4$ (**c**): $GeTe_2$

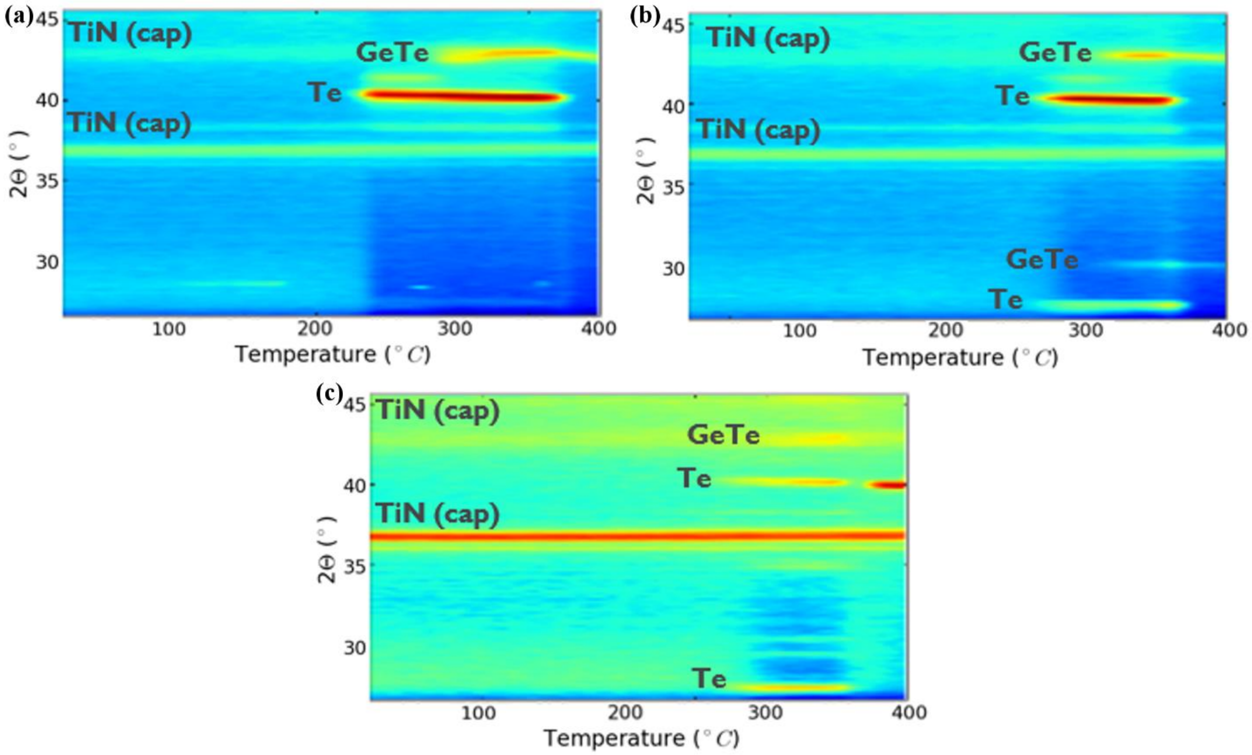

Figure S8. IS-XRD spectra for the compositions used in the second route (Si doping). (**a**): 5%Si:$GeTe_6$ (**b**): 10%Si:$GeTe_6$ (**c**): 15%Si:$GeTe_6$

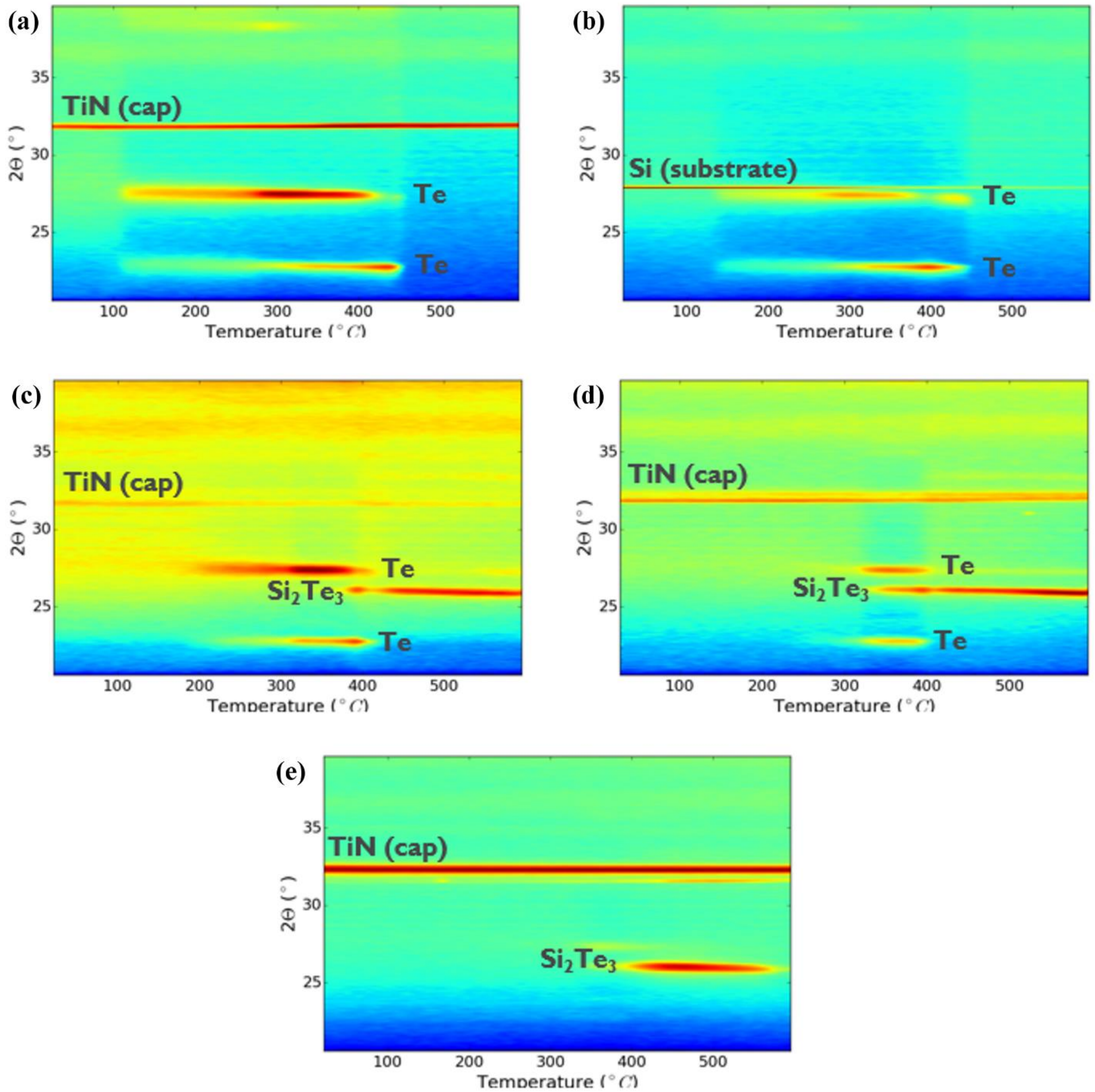

Figure S9. IS-XRD spectra for the compositions used in the third route (Si-Te binary system). (**a**): $SiTe_6$ (**b**): $SiTe_4$ (**c**): $SiTe_3$ (**d**): $SiTe_2$ (**e**): SiTe

* alin.velea@infim.ro

References

1. Jellison G. E. & Modine F. A. Parameterization of the optical functions of amorphous materials in the interband region. *Appl. Phys. Lett.* **69**, 371-373 (1996).

2. Orava J. *et al*. Optical properties and phase change transition in $Ge_2Sb_2Te_5$ flash evaporated thin films studied by temperature dependent spectroscopic ellipsometry. *J. Appl. Phys.* **104**, 043523 (2008).

3. Jiang, H. *et al*. Preparation and characterization of $GeTe_4$ thin films as a candidate for phase change memory applications. *J. Appl. Phys.* **109**, 066104 (2011).

4. Savage J. A. Glass forming region and DTA survey in the Ge-As-Te memory switching glass system. *J. Mater. Sci.* **6**, 964-968 (1971).

5. Savage J. A. Glass-forming region and DTA survey of some glasses in the Si-Ge-As-Te threshold switching glass system. *J. Mater. Sci.* **7**, 64-67 (1972).

6. Govoreanu, B. *et al.* 10×10nm^2 Hf/HfO_x crossbar resistive RAM with excellent performance, reliability and low-energy operation, *IEDM Tech. Dig.* 31.6.1-31.6.4; 10.1109/IEDM.2011.6131652 (2011).

7. Lankhorst, M.H.R. Modelling glass transition temperatures of chalcogenide glasses. Applied to phase-change optical recording materials *J. Non-Cryst. Solids* **297**, 210-219 (2002).
